# Anterior intrapelvic approach and suprapectineal quadrilateral surface plate for acetabular fractures with anterior involvement: a retrospective study of 34 patients

**DOI:** 10.1186/s12891-021-04908-z

**Published:** 2021-12-30

**Authors:** Gianluca Ciolli, Domenico De Mauro, Giuseppe Rovere, Amarildo Smakaj, Silvia Marino, Lorenzo Are, Omar El Ezzo, Francesco Liuzza

**Affiliations:** 1 Department of Orthopaedics and Traumatology, Fondazione Policlinico Universitario A, Gemelli IRCCS Largo Agostino Gemelli, 8, Rome, Italy; 2 Università Cattolica del Sacro Cuore, Rome, Italy

**Keywords:** Acetabular fracture, Anterior column, Quadrilateral surface, Suprapectineal plate, Anterior intrapelvic approach to the acetabulum

## Abstract

**Background:**

The purpose of the study is to evaluate the use of the suprapectineal quadrilateral surface (QLS) plates associated with the anterior intrapelvic approach (AIP) to the acetabulum in the surgical treatment of acetabular fractures with anterior involvement.

**Methods:**

We did a retrospective study of patients surgically treated with QLS plates and AIP for acetabular fractures with the involvement of the anterior column, between February 2018 and February 2020, in our Hospital. The following data were recorded: mechanism of injury, the pattern of fracture, presence of other associated injuries, the time before performing the surgery, surgical approach, position on operating table, time of surgery, intraoperative bleeding, hospitalization time, intraoperative and postoperative complications. Follow-ups were performed at 1, 3, 6, 12 months, then annually. The clinical-functional outcome was assessed with the Merle d’Aubigne Postel score (MAP) modified by Matta; while the radiological outcome with the Matta Radiological Scoring System (MRSS). A Chi-square test was utilized to examine associations between parametric variables.

**Results:**

We included 34 patients, mean age 62.1, with an average follow-up of 20.7 months. The most frequent traumatic mechanism was road trauma. There were 15 isolated anterior columns and 19 associated patterns. There were 5 cases of associated visceral injuries, and 10 cases of other associated skeletal fractures. All patients were in the supine position. The surgical approach used was the AIP in all cases, with the addition of the first window of the ilioinguinal approach in 16 cases and of the Kocher-Langenbeck approach in 2 cases. The average time before performing the surgery was 8.5 days. The mean time of the surgery and the mean length of stay after surgery were 227.9 min and 8.2 days, respectively. There weren’t cases of intra-operative complications, while there were postoperative complications in 5 patients. The MRSS was judged anatomical in 26 cases, imperfect in 7 cases and poor in 1 case. The average MAP value was 15.2. We observed a significant relationship between the radiological outcome and the clinical outcome (*p* < 0.05).

**Conclusions:**

The QLS plates in association with the AIP approach represent an effective treatment strategy for the treatment of acetabular fractures with anterior involvement.

## Background

Acetabulum fractures are uncommon fractures, about 5–8 per 100,000 person-years, that have always been a challenge for orthopedic surgeons. In recent decades there has been a rise in their incidence, especially in the elderly population, after a low energy trauma [1].

Acetabular fractures in the elderly usually present involvement of the anterior column of the acetabulum [2, 3].

For the specific treatment of this fracture pattern of the acetabulum was introduced new hardware, the suprapectineal quadrilateral surface (QLS) plate, which is mainly used to fix fractures of the anterior column of the acetabulum with the involvement of the quadrilateral lamina. Extensions of indications are represented by fractures of the anterior column with posterior hemitransverse, T-type fractures, and fractures of both columns, which may require additional fixation of the posterior column with axial screws or Culemann screws [4–6].

The anterior intrapelvic approach to the acetabulum (AIP) has emerged, in the last two decades, as a promising approach for fixation of anterior acetabular fractures. The AIP approach is less invasive, with less bleeding and postoperative complications than the traditional approach, while guaranteeing good exposure of acetabular fractures with involvement of the anterior column [7, 8]. The purpose of this study is to evaluate the use of the suprapectineal QLS plates associated with the AIP approach in the surgical treatment of a series of 34 cases with acetabular fractures with predominantly anterior involvement.

Our prespecified hypothesis is that this hardware is secure and ensures stable fixation in this specific type of acetabular fracture and that the AIP approach is well tolerated by the elderly and does not present particular complications.

## Methods

We did a retrospective cohort study of all pelvic fractures surgically treated in the Department of Orthopaedics of our level II Trauma Centre, between February 2018 and February 2020, in the period preceding the Covid-19 pandemic which altered the normal working activity of our Hospital [9, 10].

For the current study, we included only isolated anterior column fractures and associated patterns involving the anterior column according to Judet and Letournel classification, over 18 years of age, treated with open reduction and internal fixation using the AIP approach and suprapectineal QLS plate (PRO - Pelvis and Acetabulum System, Stryker, Kalamazoo, MI, USA) with a follow-up more than 1 year. Exclusion criteria were open fractures of the acetabulum, pathological fractures and fractures presenting after 1 month of injury [11].

All patients were examined before surgery with anteroposterior pelvis X-ray view, Judet X-ray views (obturator oblique view and iliac oblique view), and thin-slice CT with multi-planar reconstructions [12].

The following peri-operative data were taken into consideration: mechanism of injury, the pattern of fracture according to the Judet and Letournel classification, presence of other associated visceral injuries or skeletal fractures, the time before performing the surgery, surgical approach, the position adopted by the patient on the operating table, time of surgery, hospitalization time, intraoperative and postoperative complications. The surgery objectives were to obtain the anatomical reduction of the fractures and to obtain a stable and strong fixation. The radiolucent carbon table was used to allow intra-operative radiological visualization without interference.

The AIP approach is a minimally invasive intrapelvic and extraperitoneal approach that can expose the anterior column, the pelvic brim, the quadrilateral lamina and the medial portion of the posterior column. The AIP approach does not require dissection of the inguinal ligament, unlike the second window of the ilioinguinal approach described by Letournel and is particularly useful when a Cooper ligament repair has been performed or when the mesh was applied for a previous inguinal hernioplasty [7, 8].

The QLS plate is indicated in fractures where the anterior column is disrupted and the quadrilateral surface is comminuted and disassociated from the posterior column. It provides the simultaneous fixation of both columns [11].

Postoperative clinical and radiographic examinations were performed at 1, 3, 6, 12 months, and then annually. The quality of surgical reduction was assessed in anteroposterior pelvis X-ray view and Judet X-ray views by measuring the residual postoperative displacement and according to the radiographic criteria by the Matta Radiological Scoring System (MRSS) they were classified as anatomical, imperfect, or poor. According to the postoperative displacement measured on X-rays, the quality of the reduction can be evaluated anatomical (0–2 mm), imperfect (2–3 mm), or poor (> 3 mm) [13].

At the last follow-up, the clinical-functional evaluation was performed with the Merle d’Aubigne and Postel (MAP) score modified by Matta to precisely explore the patients’ pain, gait and mobility [14–16].

A Chi-square test was utilized to examine associations between parametric variables. We have analyzed the relationship between clinical outcome score (MAP) and radiological outcome (MRSS); and the relationship between the clinical outcome score (MAP) and the type of fracture according to Judet and Letournel classification. *P*-values < 0.05 were considered statistically significant.

All procedures performed in the current study were following the 1964 Helsinki declaration and its later amendments. Informed consent was obtained from all individual participants included in the study. The study design was approved by the Institute and School Council.

## Results

A total of 34 patients, 26 men and 8 women, were included in the study. The average age is 62.1 years (range 27–87 years). The mean follow-up was 20.7 months (range 12–36 months). Four of 34 (11.8%) patients were followed up at 3 years, 13 (38.2%) were followed up at 2 years, the remaining 17 (50%) at almost 1 year.

The traumatic mechanism was in most cases a road trauma (20 cases - 58.8%), followed by accidental fall (9 cases - 26.5%), and fall from height (5 cases - 14.7%). The 20 road injuries were specifically caused by accidents involving cars in 11 cases, motorcycles in 6 cases and bicycles in 3 cases. Accidental falls in all cases concerned elderly people (age over 65) while height drop (falls from a height greater than 1.5 m) occurred in the workplace.

The fractures were classified according to the Judet and Letournel classification. In all cases, there was an involvement of the anterior column, 15 isolated anterior columns (44.1%), and 19 associated patterns (55.9%), in particular, 10 (29.4%) anterior column with posterior hemitransverse, 5(14.7%) T-type, and 4 both columns (11.8%).

We have had 5 cases with associated visceral injuries, in particular 3 urinary tract and genital injuries and 2 abdominal injuries. We had 10 cases of associated skeletal fractures, in particular 6 lower limb fractures (3 femurs, 2 tibias, 1 calcaneus), and 4 upper limb fractures (2 scapulae, 1 humerus, 1 metacarpal bone). (Table 1).

**Table 1:** Patient demographics and injury characteristics

Patients	Pattern of fracture	Fracture mechanism	Other injuries
1	Anterior column	Accidental fall	
2	Anterior column	Road trauma (motorcycle)	Urogenital injury
3	Anterior column	Fall form height	
4	Anterior column with posterior hemitransverse	Fall form height	
5	Anterior column with posterior hemitransverse	Road trauma (car)	Femur fracture
6	Anterior column with posterior hemitransverse	Road trauma (car)	
7	T- type	Accidental fall	Scapula fracture
8	Anterior column	Accidental fall	
9	T- type	Road trauma (motorcycle)	Femur fracture
10	Both columns	Road trauma (motorcycle)	Urogenital injury
11	Anterior column with posterior hemitransverse	Accidental fall	
12	Both columns	Road trauma (bicycle)	Spleen injury
13	Anterior column	Road trauma (car)	
14	T- type	Accidental fall	Tibia fracture
15	Anterior column with posterior hemitransverse	Road trauma (car)	Epatic injury
16	Anterior column with posterior hemitransverse	Accidental fall	
17	Both columns	Fall form height	
18	Anterior column	Road trauma (motorcycle)	Matacarpal bone fracture
19	Anterior column with posterior hemitransverse	Road trauma (car)	
20	Anterior column	Road trauma (motorcycle)	Scapula fracture
21	Anterior column	Accidental fall	
22	T- type	Road trauma (car)	Humerus fracture
23	Anterior column	Fall form height	
24	Anterior column	Road trauma (motorcycle)	Urogenital injury
25	Anterior column	Accidental fall	
26	Anterior column with posterior hemitransverse	Road trauma (car)	Femur fracture
27	Both columns	Fall form height	
28	Anterior column with posterior hemitransverse	Accidental fall	
29	Anterior column	Road trauma (car)	Tibia fracture
30	Anterior column	Accidental fall	
31	Anterior column	Road trauma (bicycle)	
32	T- type	Road trauma (bicycle)	
33	Anterior column	Road trauma (car)	Calcaneus fracture
34	Anterior column	Road trauma (car)	

**Fig. 1:**
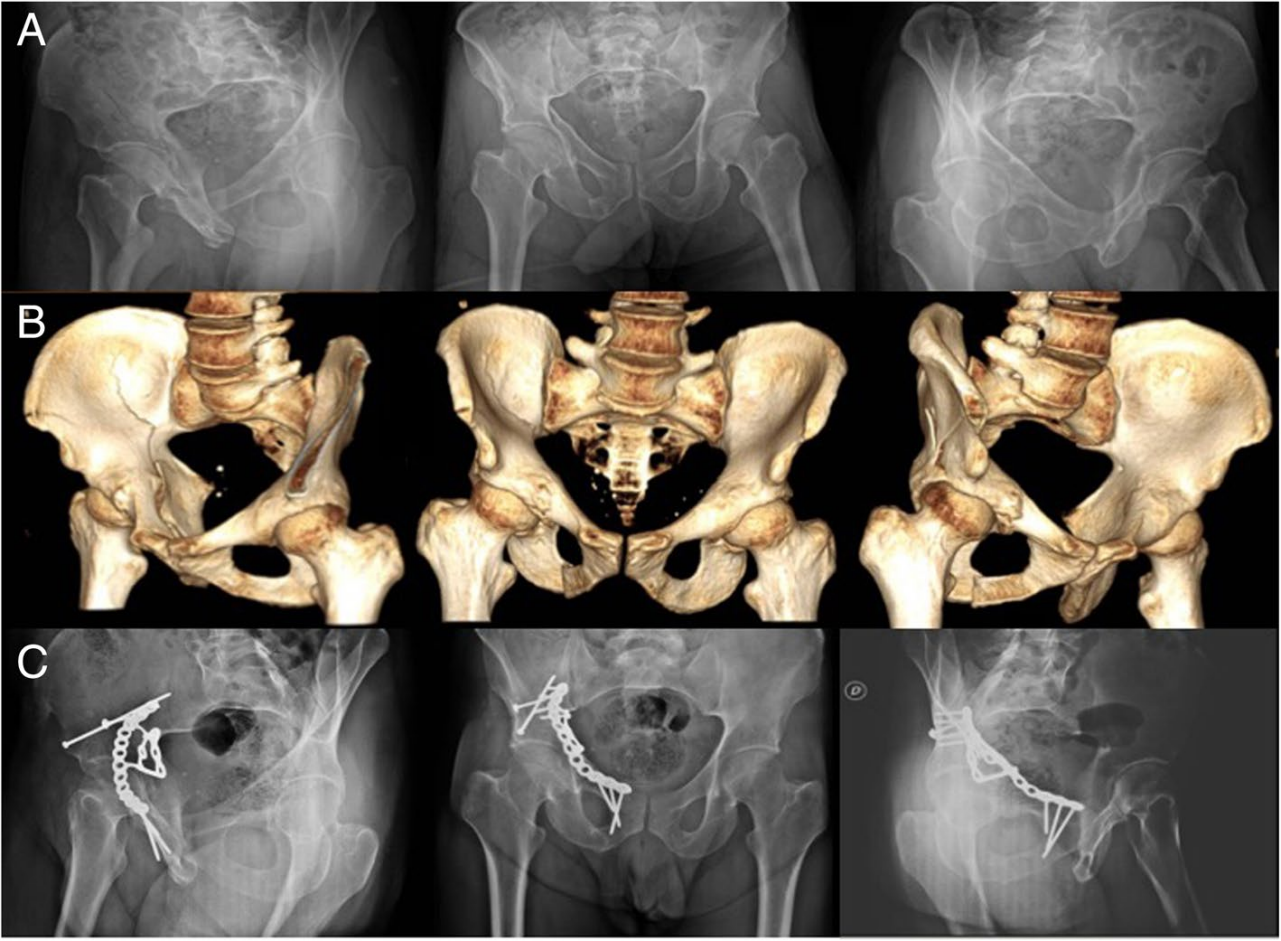
A case of a patient with both columns fracture pattern according to the Judet and Letournel classification of the right acetabulum. It was pre-operative examined with anteroposterior pelvis X-ray view and Judet X-ray views (obturator oblique view and iliac oblique view) (**A**), and thin-slice CT with 3D reconstructions (**B**). One-year radiographic follow-up with anteroposterior pelvis X-ray view and Judet X-ray views (**C**). Internal fixation was performed using the AIP approach and the QLS suprapectineal plate. Also, were used a percutaneous screw inserted with the cannulae of the pelvis pro system and an LC2 screw in the supraacetabular corridor

All patients were operated on the supine position. The surgical approach used was the AIP approach in all cases. In 16 cases (47%) is also used the first window of the ilioinguinal approach, to place axial screws in the posterior column of the acetabulum. In 2 cases (5.9%) a second-time surgery in the prone position was required, in the same surgical procedure, using the Kocher-Langenbeck approach to reduce and fix the posterior column of the acetabulum, because it was not possible to achieve with an anterior approach. (Fig. 1).

The average time before performing the surgery was 8.5 days (range 3–20 days). The mean time of the surgery and the mean length of stay after surgery were 227.9 min (range 184–358 min) and 8.2 days (range 4–17), respectively. There have been no cases of intra-operative complications. (Table 2).

**Table 2:** Surgery characteristics

Patients	Time before surgery (days)	Table Position	Surgical approach	Time of surgery (minutes)	Hospitalization time (days)
1	7	Supine	Anterior intrapelvic approach to the acetabulum	222	9
2	5	Supine	AIP + first window of the ilioinguinal approach	234	6
3	14	Supine	Anterior intrapelvic approach to the acetabulum	198	8
4	6	Supine	AIP + first window of the ilioinguinal approach	251	6
5	20	Supine	AIP + first window of the ilioinguinal approach	248	10
6	6	Supine	Anterior intrapelvic approach to the acetabulum	184	4
7	13	Supine	Anterior intrapelvic approach to the acetabulum	208	7
8	14	Supine	Anterior intrapelvic approach to the acetabulum	238	9
9	7	Supine	Anterior intrapelvic approach to the acetabulum	225	9
10	11	Supine + prone	AIP + first window of the ilioinguinal approach + Kocher-Langenbeck approach	358	6
11	4	Supine	AIP + first window of the ilioinguinal approach	206	7
12	10	Supine + prone	AIP + first window of the ilioinguinal approach + Kocher-Langenbeck approach	300	13
13	7	Supine	Anterior intrapelvic approach to the acetabulum	230	6
14	17	Supine	Anterior intrapelvic approach to the acetabulum	190	8
15	5	Supine	AIP + first window of the ilioinguinal approach	240	7
16	9	Supine	AIP + first window of the ilioinguinal approach	280	7
17	6	Supine	AIP + first window of the ilioinguinal approach	231	4
18	7	Supine	Anterior intrapelvic approach to the acetabulum	244	7
19	10	Supine	AIP + first window of the ilioinguinal approach	226	8
20	7	Supine	Anterior intrapelvic approach to the acetabulum	190	9
21	9	Supine	Anterior intrapelvic approach to the acetabulum	207	12
22	7	Supine	Anterior intrapelvic approach to the acetabulum	196	8
23	11	Supine	Anterior intrapelvic approach to the acetabulum	234	11
24	4	Supine	Anterior intrapelvic approach to the acetabulum	196	14
25	9	Supine	Anterior intrapelvic approach to the acetabulum	217	10
26	6	Supine	AIP + first window of the ilioinguinal approach	210	5
27	16	Supine	AIP + first window of the ilioinguinal approach	214	5
28	7	Supine	Anterior intrapelvic approach to the acetabulum	220	9
29	6	Supine	AIP + first window of the ilioinguinal approach	256	17
30	9	Supine	AIP + first window of the ilioinguinal approach	247	12
31	3	Supine	AIP + first window of the ilioinguinal approach	260	7
32	7	Supine	Anterior intrapelvic approach to the acetabulum	195	5
33	4	Supine	Anterior intrapelvic approach to the acetabulum	189	6
34	6	Supine	Anterior intrapelvic approach to the acetabulum	203	7

*AIP* Anterior intrapelvic approach to the acetabulum

We observed postoperative complications in 5 patients (14.7%): one case of deep infection required surgical removal of the hardware; one case of intra-articular penetration of a screw from the anterior column requiring screw removal and 3 cases of deep venous thrombosis (1 posterior tibial vein, 1 superficial femoral vein, and 1 femoral vein) treated with low molecular weight heparin.

At the one-year radiographic follow-up, delayed union or malunion were not recorded.

The radiological outcome, assessed according to Matta’s radiological criteria, was judged anatomical in 26 cases (76.5%), imperfect in 7 cases (20.6%) and poor in 1 case (2.9%).

The average MAP value was 15.2 (range 9–18); evaluated excellent in 6 cases (17.6%) good in 16 cases (47%), fair in 10 cases (29.4%) and poor in 2 cases (5.9%). (Table 3).

**Table 3:** Patient post-operative complications, outcomes and follow-up

Patients	Post-operative complications	MAP	MRSS	Follow-up (months)
1		POOR (9)	POOR	36
2		GOOD (17)	ANATOMICAL	24
3		GOOD (16)	ANATOMICAL	24
4		GOOD (15)	ANATOMICAL	24
5		GOOD (15)	ANATOMICAL	18
6		GOOD (16)	ANATOMICAL	24
7		FAIR (14)	ANATOMICAL	24
8	Intra-articular penetration of a screw	FAIR (13)	IMPERFECT	24
9		EXCELLENT (18)	ANATOMICAL	24
10		GOOD (16)	ANATOMICAL	18
11		GOOD (16)	ANATOMICAL	18
12		EXCELLENT (18)	ANATOMICAL	18
13		GOOD (16)	ANATOMICAL	24
14	Deep vein thrombosis (superficial femoral vein)	POOR (11)	IMPERFECT	24
15		EXCELLENT (18)	ANATOMICAL	18
16		GOOD (15)	ANATOMICAL	24
17		GOOD (16)	ANATOMICAL	12
18		EXCELLENT (18)	ANATOMICAL	12
19	Deep infection of the hardware	FAIR (13)	IMPERFECT	18
20		GOOD (17)	ANATOMICAL	12
21		GOOD (16)	ANATOMICAL	12
22		GOOD (17)	ANATOMICAL	36
23		FAIR (14)	ANATOMICAL	24
24		EXCELLENT (18)	ANATOMICAL	18
25		FAIR (14)	IMPERFECT	24
26	Deep vein thrombosis (posterior tibial vein)	GOOD (15)	ANATOMICAL	36
27		FAIR (13)	IMPERFECT	12
28		GOOD (16)	ANATOMICAL	36
29		FAIR (14)	ANATOMICAL	12
30	Deep vein thrombosis (femoral vein)	FAIR (13)	IMPERFECT	18
31		GOOD (17)	ANATOMICAL	18
32		FAIR (13)	IMPERFECT	12
33		EXCELLENT (18)	ANATOMICAL	12
34		FAIR (13)	ANATOMICAL	12

*MAP* Merle d’Aubigne and Postel score, *MRSS* Matta Radiological Scoring System

We observed a significant relationship between the clinical outcome and the radiological outcome (*p* < 0.05), while we did not observe significant relationships between the clinical outcome and the type of fracture (*p* > 0.05).

## Discussion

Fractures of the pelvis are uncommon fractures, with bimodal distribution in the population, caused by high-energy trauma in the young, like motor vehicle collision, and by low-energy trauma in the elderly, as falling on the same level. In recent decades there has been a rise in the incidence of these fractures, thanks to the increase in the survival rates of the most critical patients and to the improvement of emergency care [1, 2].

The treatment of acetabulum fractures needs an open approach for anatomical reduction and fixation of the fragment, also in the elderly [13, 14, 17–19].

In older age there is an increase of specific patterns of fracture involving the anterior acetabular structures: anterior column, quadrilateral lamina fracture, medial dislocation of the femoral head, and roof impaction (with the specific Gull sign), differ from those in younger patients [20, 21].

The anterior column is formed from a combination of the ilium and pubic bones. Anterior column fracture is an elementary fracture according to the Judet and Letournel classification that could be isolated or involved in an associated pattern of fractures with posterior hemitransverse, T-type, and both columns, that require a specific fixation technique [22].

A traditional fixation method of these fractures uses lag screws over a suprapectineal plate through the ilioinguinal approach described by Letournel. In addition, an infrapectineal plate could be used to provide a buttress effect against the protrusion of the femur into the pelvis. The combination of a standard pelvic brim plate with lag screws and an infrapectineal plate supporting the quadrilateral lamina resulted in a better fixation construct and provide better stability with the advantages in the prevention of construct failure in situations in which significant lateral to medial force is applied, such as patient falls on homolateral hip [23–26].

In recent years, new alternative hardware has been introduced for the treatment of anterior acetabular fractures, the suprapectineal QLS plate, which is an anatomic preshaped plate, that represents a valid alternative to the infrapectineal plates, providing a better dynamic buttress effect to the comminuted fragments of the quadrilateral lamina, and preventing the medial subluxation of the femoral head. This hardware allows the simultaneous fixation of the anterior column, with the suprapectinal portion of the plate, and the quadrilateral lamina, with the infrapectineal portion; so are useful in the treatment of different fracture patterns: anterior column, anterior column with posterior hemitransverse, T- type and both columns. They have been specifically designed to prevent secondary medial subluxation of the femoral head, especially in elderly patients with reduced partial load capacity; moreover, screws placed in the quadrilateral lamina extension were not in danger for intraarticular placement as demonstrated in CT scans [4, 27, 28].

In the literature is reported a great variability of osteosynthesis for the treatment of fractures of the anterior column of the acetabulum with the involvement of the quadrilateral surface, without consensus in the choices.

Boni G et al. use a suprapectineal plate with the addition of a stainless-steel locking calcaneal plate, through the modified Stoppa approach, to fix quadrilateral lamina; while Farid YR et al. proposed a cerclage wire-plate composite fixation with an extraosseous cerclage and a reconstruction plate over the pelvic brim [29, 30].

Another possible treatment uses a 3.5 mm or 4.5 mm reconstruction plate on the pelvic brim partially protruding medially into the true pelvis and in addiction one or more buttress screws inserted through the plate holes, on the outside surface of the quadrilateral surface close to the edge of the pelvic brim [31].

Kulkarni et al. described the treatment of comminuted quadrilateral plate fractures of the acetabulum using a modified Stoppa approach and a spring buttressing plate with good scores in clinical and radiological outcome at 1 year follow up [32].

In our experience, the AIP approach is associated with the suprapectineal QLS plates. In some cases, if the fracture of the anterior column is high or very displaced can be combined with the first window, the lateral one, of the ilioinguinal approach described by Letournel [4, 5, 33].

In our series, we were able to achieve an anatomical reduction with a postoperative displacement < 1 mm in 26 of 34 cases.

Our clinical and radiographic results are comparable to those obtained by other authors. Archdeacon et al., using a combination of suprapectineal and infrapectineal plates, found an average MAP score of 16 and an excellent MRSS in 15, good in 3, poor in 3; while Tosounidis et al., using a quadrilateral plate reconstruction with a buttress plate through the ilioinguinal approach obtain in 30 patients an MRSS excellent in 11, good in 9, fair in 5 and poor in 5; while the overall functional score was excellent in 17, good in 4, fair in 6 and poor in 3 cases [23, 26].

This shows that the QLS plate in combination with the AIP approach makes it possible to obtain a valid endopelvic exposure of the fracture that allows for a good reduction and consequently a stable fixation.

At the last clinical follow-up examination, all patients reported a good functional restore with no or mild pain and no or slight hip stiffness not particularly affecting their quality of life, but those with an anatomical reduction had an excellent clinical outcome.

The AIP approach utilizing the anatomical-preshaped suprapectineal plate allows anatomic or at least imperfect fracture reduction, according to Matta’s radiological criteria, in 97% of cases of our study.

In our experience, the plates have shown an excellent anti-protrusion effect of the femoral head and quadrilateral lamina, without a record of delayed union or malunion.

This procedure with the combination of a specific anesthesiology technique as the supra-inguinal fascia iliaca compartment block has become the standard procedure in our departments for the fracture of the acetabulum that involves the anterior column [34].

The strength of our study is that to our knowledge there is no case series in the literature of patients treated with the combined use of the AIP approach and QLS plate.

Limitations of the study are the small number of cases, different follow-up times, lack of a case-control treatment with an alternative fixation method and short-term follow-up.

## Conclusions

From the literature analyzed and with our experience, it is possible to confirm that the suprapectineal QLS plates represent an effective and safe system of fixation for the treatment of acetabulum fractures involving the anterior column. The AIP approach to the acetabulum is demonstrated to be a safe, effective, and feasible alternative to the traditional ilioinguinal approach for acetabulum fractures which require an anterior approach.

## Data Availability

The datasets used and/or analyzed during the current study are available from the corresponding author on reasonable request.

## Abbreviations

QLS: Quadrilateral surface plate
AIP: Anterior intrapelvic approach to the acetabulum
MAP: Merle d’Aubigne and Postel score
MRSS: Matta Radiological Scoring System

## References

[1] Melhem E, Riouallon G, Habboubi K, Gabbas M, Jouffroy P (2020). Epidemiology of pelvic and acetabular fractures in France. Orthop Traumatol Surg Res.

[2] Rinne PP, Laitinen MK, Kannus P, Mattila VM (2020). The incidence of pelvic fractures and related surgery in the Finnish adult population: a nationwide study of 33,469 patients between 1997 and 2014. Acta Orthop.

[3] Liuzza F, Silluzio N, Florio M, El Ezzo O, Cazzato G, Ciolli G, Perisano C, Maccauro G (2019). Comparison between posterior sacral plate stabilization versus minimally invasive transiliac-transsacral lag-screw fixation in fractures of sacrum: a single-Centre experience. Int Orthop.

[4] Kistler BJ, Smithson IR, Cooper SA, Cox JL, Nayak AN, Santoni BG, Sagi HC (2014). Are quadrilateral surface buttress plates comparable to traditional forms of transverse acetabular fracture fixation?. Clin Orthop Relat Res.

[5] Chen K, Yang F, Yao S, Xiong Z, Sun T, Guo X (2020). Biomechanical comparison of different fixation techniques for typical acetabular fractures in the elderly: the role of special quadrilateral surface buttress plates. J Bone Joint Surg Am.

[6] Schmidt W, LiArno S, Khlopas A, Petersik A, Mont MA (2018). Stryker Orthopaedic modeling and analytics (SOMA): a review. Surg Technol Int.

[7] Trikha V, Das S, Aruljothi V, Chowdhury B (2020). prospective evaluation of outcome of acetabular fractures managed by anterior intrapelvic approach. Indian J Orthop.

[8] Sagi HC, Afsari A, Dziadosz D (2010). The anterior intra-pelvic (modified rives-stoppa) approach for fixation of acetabular fractures. J Orthop Trauma.

[9] Ciolli G, Caviglia D, Vitiello C, Lucchesi S, Pinelli C, De Mauro D, Smakaj A, Rovere G, Meccariello L, Camarda L, Maccauro G, Liuzza F (2021). Navigated percutaneous screw fixation of the pelvis with O-arm 2: two years' experience. Med Glas (Zenica)..

[10] De Mauro D, Rovere G, Smimmo A, Meschini C, Mocini F, Maccauro G, Falez F, Liuzza F, Ziranu A (2020). COVID-19 pandemic: management of patients affected by SARS-CoV-2 in Rome COVID Hospital 2 Trauma Centre and safety of our surgical team. Int Orthop.

[11] Letournel E (1980). Acetabulum fractures: classification and management. Clin Orthop Relat Res.

[12] Visutipol B, Chobtangsin P, Ketmalasiri B, Pattarabanjird N, Varodompun N (2000). Evaluation of Letournel and Judet classification of acetabular fracture with plain radiographs and three-dimensional computerized tomographic scan. J Orthop Surg (Hong Kong).

[13] Matta JM (1996). Fractures of the acetabulum: accuracy of reduction and clinical results in patients managed operatively within three weeks after the injury. J Bone Joint Surg Am.

[14] Matta JM, Mehne DK, Roffi R. Fractures of the acetabulum. Early results of a prospective study. Clin Orthop Relat Res. 1986;(205):241–50 PMID: 3698383. 3698383

[15] Meena UK, Tripathy SK, Sen RK, Aggarwal S, Behera P (2013). Predictors of postoperative outcome for acetabular fractures. Orthop Traumatol Surg Res.

[16] Muzii VF, Rollo G, Rocca G, Erasmo R, Falzarano G, Liuzza F, Bisaccia M, Pica G, Franzese R, Meccariello L (2021). Radiographic and functional outcome of complex acetabular fractures: implications of open reduction in spinopelvic balance, gait and quality of life. Med Glas (Zenica).

[17] Liuzza F, Capasso L, Florio M, Mocini F, Masci G, Cazzato G, Ciolli G, Silluzio N, Maccauro G (2018). Transiliosacral fixation using the O-ARM2® and STEALTHSTATION® navigation system. J Biol Regul Homeost Agents.

[18] Florio M, Capasso L, Olivi A, Vitiello C, Leone A, Liuzza F (2020). 3D - Navigated percutaneous screw fixation of pelvic ring injuries - a pilot study. Injury.

[19] Rovere G, Perna A, Meccariello L, De Mauro D, Smimmo A, Proietti L, et al. Epidemiology and aetiology of male and female sexual dysfunctions related to pelvic ring injuries: a systematic review. Int Orthop. 2021. 10.1007/s00264-021-05153-8 Epub ahead of print. PMID: 34378143. 10.1007/s00264-021-05153-8 PMC8514382 34378143

[20] Butterwick D, Papp S, Gofton W, Liew A, Beaulé PE (2015). Acetabular fractures in the elderly: evaluation and management. J Bone Joint Surg Am.

[21] Antell NB, Switzer JA, Schmidt AH (2017). Management of Acetabular Fractures in the elderly. J Am Acad Orthop Surg.

[22] White G, Kanakaris NK, Faour O, Valverde JA, Martin MA, Giannoudis PV (2013). Quadrilateral plate fractures of the acetabulum: an update. Injury..

[23] Archdeacon MT, Kazemi N, Collinge C, Budde B, Schnell S (2013). Treatment of protrusio fractures of the acetabulum in patients 70 years and older. J Orthop Trauma.

[24] Gillispie GJ, Babcock SN, McNamara KP, Dimoff ME, Aneja A, Brown PJ, Carroll EA (2017). Biomechanical comparison of Intrapelvic and Extrapelvic fixation for Acetabular fractures involving the quadrilateral plate. J Orthop Trauma.

[25] Qureshi AA, Archdeacon MT, Jenkins MA, Infante A, DiPasquale T, Bolhofner BR (2004). Infrapectineal plating for acetabular fractures: a technical adjunct to internal fixation. J Orthop Trauma.

[26] Tosounidis TH, Gudipati S, Panteli M, Kanakaris NK, Giannoudis PV (2015). The use of buttress plates in the management of acetabular fractures with quadrilateral plate involvement: is it still a valid option?. Int Orthop.

[27] Gras F, Marintschev I, Grossterlinden L, Rossmann M, Graul I, Hofmann GO, Rueger JM, Lehmann W (2017). The anterior Intrapelvic approach for Acetabular fractures using approach-specific instruments and an anatomical-Preshaped 3-dimensional Suprapectineal plate. J Orthop Trauma.

[28] Egli RJ, Keel MJB, Cullmann JL, Bastian JD (2017). Secure screw placement in management of acetabular fractures using the suprapectineal quadrilateral buttress plate. Biomed Res Int.

[29] Boni G, Pires RE, Sanchez GT, Dos Reis FB, Yoon RS, Liporace FA (2019). Use of a stainless steel locking calcaneal plate for quadrilateral plate buttress in the treatment of acetabular fractures. Eur J Orthop Surg Traumatol.

[30] Farid YR (2010). Cerclage wire-plate composite for fixation of quadrilateral plate fractures of the acetabulum: a checkrein and pulley technique. J Orthop Trauma.

[31] Karim MA, Abdelazeem AH, Youness M, El Nahal WA (2017). Fixation of quadrilateral plate fractures of the acetabulum using the buttress screw: a novel technique. Injury..

[32] Kulkarni C, Sen A, Jose J. Outcome analysis of surgical management of comminuted quadrilateral plate acetabulum fractures. Int J Res Orthop. 2019;5. 883. 10.18203/issn.2455-4510 IntJResOrthop20193829.

[33] Nikolopoulos FV, Tzoras NT (2019). (2019) the advantages of Stoppa approach-ilioinguinal modification, for surgical treatment of the acetabulum fractures with the traditional plate and the new anatomical Suprapectineal plate system. J Orthop Case Rep.

[34] Vergari A, Frassanito L, Tamburello E, Nestorini R, Sala FD, Lais G, Ciolli G, Liuzza F (2020). Supra-inguinal fascia iliaca compartment block for postoperative analgesia after Acetabular fracture surgery. Injury..

